# Signature of adaptive evolution in olfactory receptor genes in Cory’s Shearwater supports molecular basis for smell in procellariiform seabirds

**DOI:** 10.1038/s41598-019-56950-6

**Published:** 2020-01-17

**Authors:** Mónica C. Silva, Marcus Chibucos, James B. Munro, Sean Daugherty, M. Manuela Coelho, Joana C. Silva

**Affiliations:** 1 cE3c - Centre for Ecology, Evolution and Environmental Changes, Faculdade de Ciências, Universidade de Lisboa, 1749-016 Lisboa, Portugal; 2 Institute for Genome Sciences, University of Maryland School of Medicine, Baltimore, USA; 3 Department of Microbiology and Immunology, University of Maryland School of Medicine, Baltimore, USA

**Keywords:** Evolutionary genetics, Molecular evolution

## Abstract

Olfactory receptors (ORs), encoded by the largest vertebrate multigene family, enable the detection of thousands of unique odorants in the environment and consequently play a critical role in species survival. Here, we advance our knowledge of OR gene evolution in procellariiform seabirds, an avian group which relies on the sense of olfaction for critical ecological functions. We built a cosmid library of Cory’s Shearwater (*Calonectris borealis*) genomic DNA, a model species for the study of olfaction-based navigation, and sequence OR gene-positive cosmid clones with a combination of sequencing technologies. We identified 220 OR open reading frames, 20 of which are full length, intact OR genes, and found a large ratio of partial and pseudogenes to intact OR genes (2:1), suggestive of a dynamic mode of evolution. Phylogenetic analyses revealed that while a few genes cluster with those of other sauropsid species in a *γ* (*gamma*) clade that predates the divergence of different avian lineages, most genes belong to an avian-specific *γ*-c clade, within which sequences cluster by species, suggesting frequent duplication and/or gene conversion events. We identified evidence of positive selection on full length *γ-*c clade genes. These patterns are consistent with a key role of adaptation in the functional diversification of olfactory receptor genes in a bird lineage that relies extensively on olfaction.

## Introduction

The sense of olfaction is used by vertebrates to process information about their surroundings, playing a critical role in survival and reproduction as it facilitates the recognition of suitable food, predators and prey, mates, and territories^1,2^. Odour perception begins with the binding of odorant molecules to G protein-coupled receptors (GPCRs) primarily expressed in the nasal olfactory epithelium^3^. These receptors, encoded by a complex multi-gene family containing large numbers of olfactory receptor (OR) genes^4^, allow discrimination of a vast array of unique odorants.

**Figure 1:**
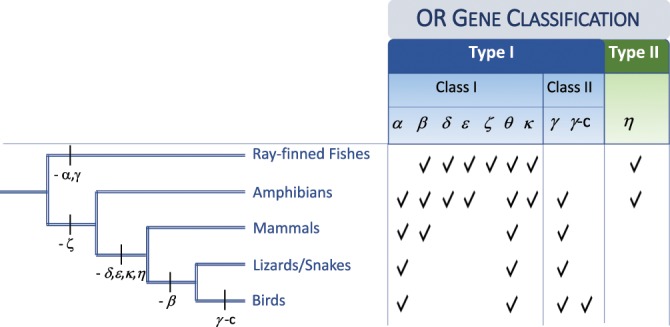
Classification and schematic distribution of olfactory receptor genes in major vertebrate lineages.

Phylogenetic analyses of olfactory receptor genes in model vertebrates enabled the identification of nine different monophyletic classes of receptors (*α, β, δ, ε, γ, ξ, η, θ* and *κ*) divided into Type I and Type II^5,6^ (Fig. 1). Type I is the most diverse, and includes Class I (*α, β, δ, ε, ξ* and *θ* groups) and Class II (*γ*). Particularly the *α* and *γ* groups have experienced extremely large copy number expansions and because they occur in tetrapods they are thought to mediate the recognition of airborne odorants^5,7^ (Fig. 1). The remaining Type I and Type II (which only includes group *η*) are mainly present in fish and amphibian genomes and are thought to detect water-soluble odorants^5^. The avian species surveyed in greater genomic depth (the chicken, *Gallus gallus* and the zebra finch *Taeniopygia guttata*) mostly have OR genes belonging to group *γ*, but also a few *α* and *θ*^6,8,9^.

Comparative studies have shown that OR gene copy number can vary by several orders of magnitude among vertebrate groups, and even among closely related lineages^10–12^. Although the proportion of OR pseudogenes is characteristically high (20–60%^11^), the relative numbers of intact, pseudo- (with an in-frame stop codon) and partial genes (without a start and/or stop codons) can also vary substantially among and within species^11^. Multi-gene families characterized by this diversity pattern follow a birth-and-death model of evolution^13,14^, according to which gene copy number variation emerges due to repeated bouts of duplication, deletion and gene conversion events^14^. While gene duplication occurs primarily due to unequal crossing over^5^, retention of duplicated genes may be facilitated by natural selection if a different and/or a larger number of genes confers a selective advantage by generating a more diverse receptor repertoire^9,10,15–17^. The striking variation in OR diversity patterns has been related to sensory trade-offs and lineage-specific ecological adaptation to new niches^5,6,10,12,18–21^. Despite this extraordinary gene diversity, OR gene structure is fairly conserved. Functional genes are approximately 1Kb long and generally lack introns. Based on patterns of variation and computational modeling in model organisms, the putative ligand binding sites are mainly located between the third and seventh transmembrane (TM) helical domains^22,23^.

Although it is now widely accepted that birds are able to recognize and act upon particular odours^2,24^, research on avian OR genes has been mostly based on *G. gallus*. The first survey in a taxonomically diverse group of nine bird species found a striking pattern of OR gene diversity, characterized by the presence of two distinct *γ* clades^25^. Genes clustered with putative orthologs from other species in one clade, suggesting that this *γ* sub-group was already present in the common ancestor of the different bird lineages^25^. In contrast, in the most abundant *γ-c* clade (also referred to as family 14^6^), genes clustered by species, consistent with ubiquitous duplication events post-dating the divergence of different bird species. This expansion was later confirmed in the chicken and zebra finch genomes but found missing in the green anole and was thus hypothesized to be a unique feature of avian genomes^8,9^. A genomic survey of two falcon species found a small number of intact OR genes and a missing *γ-c* clade, possibly related to a foraging strategy more dependent on vision than olfaction^26^. A higher proportion of pseudogenes was found in penguins than in Procellariiformes, their sister group, a pattern that was also interpreted as resulting from a higher reliance on (underwater) vision for foraging^27^.

Seabirds in the avian order Procellariiformes, which includes albatrosses, petrels and shearwaters, have one of the largest relative olfactory bulb to brain size (OB) ratio of all birds^28,29^, and use the sense of smell in homing, foraging and navigation^30–32^. They forage day and night in the open ocean by building cognitive maps based on olfactory oceanic landscapes^32,33^. Despite having the largest OB ratio recorded in a group of nine species surveyed, the Snow petrel *Pagodroma nivea* was found to have amongst the fewest estimated total number of OR genes (n = 212), and among the surveyed genes, approximately 50% belong to the γ-c clade^25^. More recently, a survey of the genome of the Northern fulmar *Fulmarus glacialis* found 370 OR genes, of which 102 (only two intact) belong to the *γ-c* clade^6^. We focused on the Cory’s Shearwater *Calonectris borealis*, which is becoming a model seabird species for many ecological questions, including the importance of olfaction for homing and oceanic navigation^31–33^. Our goal was to extend the understanding of the evolution of OR genes to the Procellariiformes avian group, through their extensive characterization in *C. borealis*. Specifically, we aimed to determine 1) olfactory receptor gene diversity in *C. borealis*, 2) the ratio of full length to partial ORs and 3) whether there is evidence of lineage-specific expansion events in this species. Additionally, we aimed to find whether selection has been shaping the evolution of shearwater OR genes. This study will allow us to determine whether the evolutionary patterns observed are consistent with the hypothesis that Cory’s shearwaters rely heavily on their sense of smell for key functions such as foraging and homing, a critical contribution to the understanding of the genetic underpinnings of adaptation in this group.

## Results

### Genomic characterization of OR genes in Cory’s Shearwater

The characterization of the full genomic complement of OR gene families remains an important but challenging endeavour^34,35^. Multigene families can be reliably studied from high-quality genome references, but the combination of read lengths and the depth of coverage required make these costly and time consuming^36,37^. Conversely, short-read whole genome sequence data, while relatively inexpensive to generate, results in partial gene family sets with missing, truncated and possibly chimeric gene sequences, especially in families composed of genes with high pairwise sequence similarity or highly conserved sequence motifs^38,39^. Here, we opted for sequencing, at high depth of coverage and with a combination of sequencing technologies, a large number of long, OR- containing cosmids from a Cory’s shearwater genomic DNA library, a compromise on OR family completeness to achieve high-quality, representative gene sequences.

The most comprehensive hybrid assembly of the OR-containing cosmids had a cumulative length of 7.4 Mb. It consisted of 399 unique scaffolds with a cumulative length of 2.6 Mb and 22 K degenerate contigs totaling 4.8 Mb (Supplementary Info). The final set of *EVm* models supported by ORFs consisted of 1,501 genes encoding proteins with a mean length of 135 amino-acids (range of 75–1215 amino acids). Of these, 317 were found in the assembly scaffolds and the remaining in the degenerate contigs.

We identified 220 Cory’s shearwater full and partial length OR genes among the 1,501 gene models, ~ 60% of the OR gene number found in the genome assembly of the Northern fulmar, the only procellariidae with a draft genome available^6,40^, and 35% of the OR gene number found in the genomes of *G. gallus* and *T. gutatta*. Many additional small ORFs were discarded due to size (<75 amino acids), despite significant homology with other avian OR genes, so this is likely an underestimate of the total number of olfactory receptor genes in Cory’s shearwaters.

In total, 45 OR genes were in the main scaffolds, where ORFs ranged between 297–936 bp (with mean length ± standard deviation of 678.3 ± 249.3 bp). The remaining 175 OR genes were found in the degenerate contigs, and the ORFs ranged in length between 225–945 bp (mean ± SD 394.9 ± 118.6 bp).

### Structure and proportion of potentially functional shearwater OR genes

Of all OR genes identified, only 56 (~ 25%) were not at the edge of a contig. Of these, 20 (36%) were complete genes, 15 (27%) were pseudogenes and 21 (38%) were partial sequences (missing a start and/or stop codon and no in-frame stop codons). In all except four cases there was only one OR locus per scaffold, even though almost 80 scaffolds were above 10 Kb (Fig. S1b). In the scaffolds with more than one OR locus, ORFs were between 44 and 1,517 bp apart, and at least one was always a pseudogene. Of the 220 OR genes, 85% were annotated as putatively belonging to the *γ-c* clade (family 14^6^) (Table 1), 18 of which are complete. The remaining ORs belong to families already identified in birds and reptiles except for one gene, with highest sequence similarity to mammal genes (24-like family gene) (Table 1).

**Table 1:** Functional annotation and location of the 220 Olfactory receptor genes found in the Cory’s Shearwater genome.

Clade	Sub-family	Number of OR genes	Location	Integrity*
γ - c	*14j1-like*	73	Scaffold (9)/Degenerate (64)	9 intact, 4 partial 5 pseudogene, 55 truncated
γ - c	*14A16 -ike*	58	Scaffold (13)/Degenerate (45)	6 intact, 5 partial 5 pseudogene, 42 truncated
γ - c	*14I1-like*	7	Scaffold (2)/Degenerate (5)	5 partial, 2 truncated
γ - c	*14C36-like*	47	Scaffold (6)/Degenerate (41)	2 intact, 4 partial 3 pseudogene, 38 truncated
γ - c	*14A2-like*	1	Scaffold	1 truncated
γ	*5v1-like*	1	Scaffold	1 intact
γ	*5F1-like*	2	Scaffold	1 intact, 1 partial
γ	*24-like*	1	Degenerate	1 truncated
γ	*4a16-like*	2	Scaffold (1)/Degenerate (1)	2 truncated
γ	*6B1-like*	1	Scaffold	1 intact
γ	*13c2-like*	2	Degenerate	2 truncated
γ	*11a1-like*	1	Scaffold	1 partial
γ	*10ag1-like*	10	Scaffold (1)/Degenerate (9)	3 partial, 2 pseudogene 5 truncated
?	*unknown*	14	Degenerate	12 truncated

Genes that could not be reliable assigned to an OR sub-family with the functional annotation pipeline belong to an “unknown” family. *Genes were classified as Intact if they had start and stop codons, no premature stops or frameshift mutations; Partial if they had an incomplete coding region and were not at the edge of a contig; Pseudogene if they had the full coding region but had internal stop or frameshifts, Truncated if they were located at the edge of a contig.

**Figure 2:**
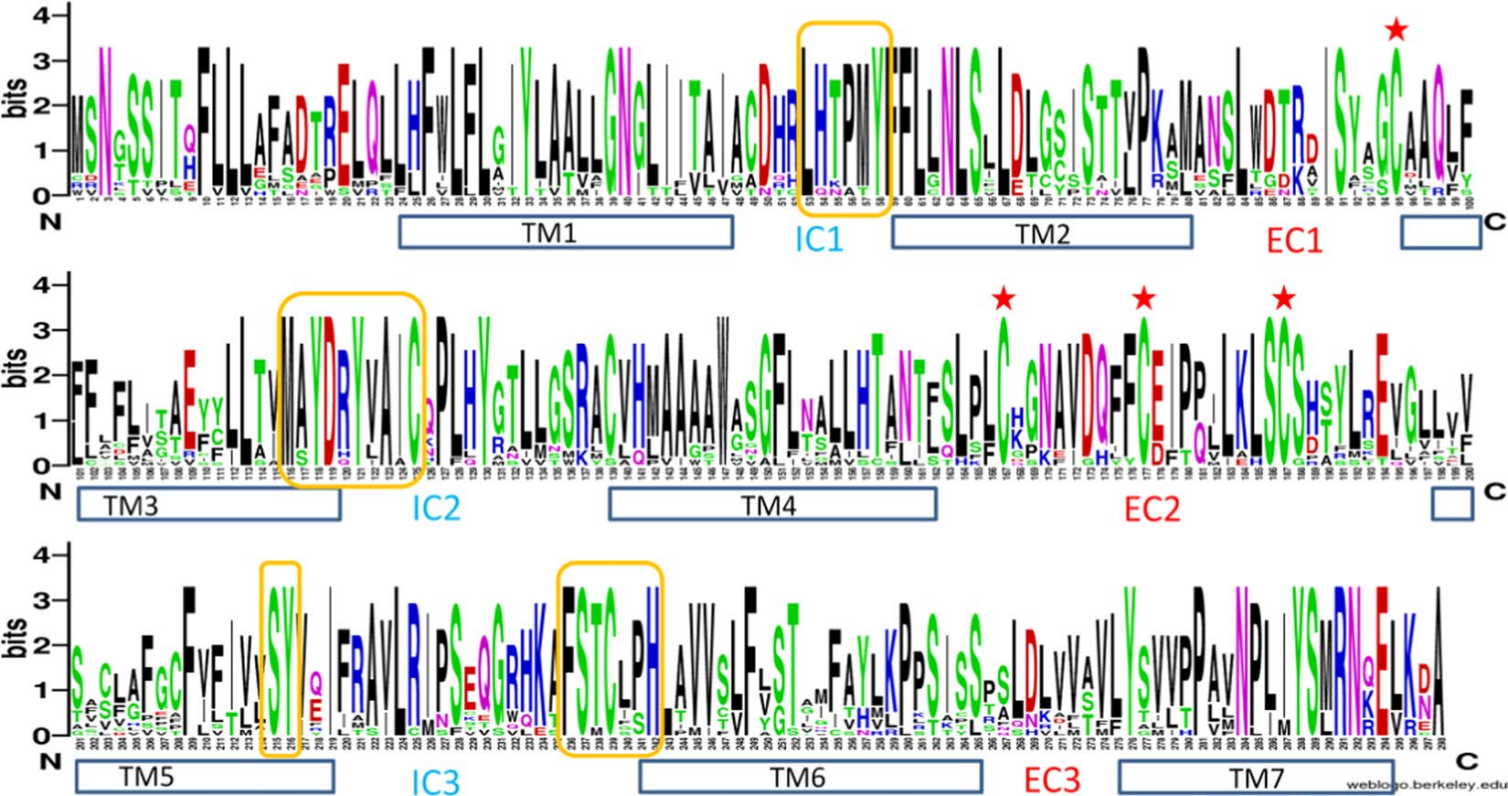
Sequence conservation in complete OR *γ-c* shearwater genes. The open reading frames of the aligned gene set (n = 17) was used to build a sequence logo. Putative location of transmembrane regions, (TM1-7), intra-cellular loops (IC1-3), extra-cellular loops (EC1-3), conserved motifs (□) and residues (✶) are shown.

Full-length *γ-c* shearwater OR genes include a single exon with a length of 312 amino acids. The logo view of the alignment suggests that the distribution of genetic variation is not uniform along the length of these genes (Fig. 2). The characteristic, conserved olfactory receptor-specific motifs are present, such as the *MAYDRYVAIC* in the transmembrane domain (TM) 3 – intracellular domain (IC) 2 boundary, the *FSTC(LP)H* at the end of IC3, and the conserved cysteine residues in the extra-cellular (EC) 1 and EC2 domains and prolines at TM7 (Fig. 2), which are important for the conformation stability of the receptor^41^. The most variable positions are found in the TM 4 and TM5 and to a lesser extent in TM3 and TM6 (Fig. 2).

### Phylogenetics of shearwater OR gene sequences

**Figure 3:**
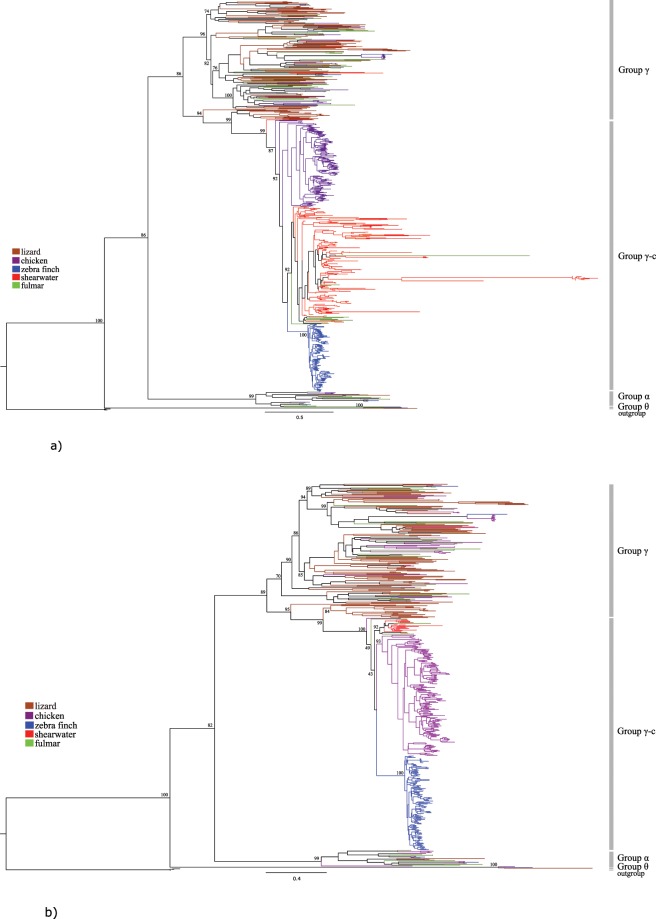
Maximum-likelihood phylogenetic analysis of sauropsid olfactory receptor genes. (**a**) Analysis includes the Cory’s Shearwater’s 220 OR genes aligned with 98 Northern fulmar genes from GenBank, 134 zebra finch genes, 214 chicken genes, and 112 green anole genes (from [8]). (**b**) Analysis using only the set of complete genes from the Cory’s Shearwater (n = 20), aligned with 44 full fulmar genes (from GenBank), 134 zebra finch genes, 214 chicken genes, and 112 green anole genes (from [8]). Both analyses are based on the JTT model as the best fitting substitution model. Outgroup sequences of the Adenosine receptor A2b are from GenBank. Nodal support values are shown for major clades.

Phylogenetic analyses suggest that there are at least two distinct OR gene clades in the shearwater (Fig. 3a), in agreement with the functional annotation. A few OR genes cluster with those of the other sauropsid species in a *γ* clade that pre-dates the divergence of the different bird lineages. However, most chicken, zebra finch, fulmar and shearwater OR genes belong to the avian specific, well-supported *γ-c* clade, within which sequences cluster by avian family (Fig. 3a). The fulmar *γ-c* genes cluster with the shearwater genes in a paraphyletic Procellariidae clade, some clustering in a fulmar specific clade, suggesting that they are younger than the divergence of the two procellariids. When all 220 sequences are included in the analysis, some shearwater and fulmar *γ-c* sequences appear to be evolving at significantly faster rates than those from the other species, as revealed by the very long branch lengths (Fig. 3a). This pattern may be due to a large number of pseudogenes evolving free of selective constraints, as it does not persist in analysis including only intact OR genes (Fig. 3b). In the analysis with the intact genes, the monophyly of the avian *γ-*c clade remains well supported as is the clade including all shearwater genes (bootstrap value of 92%).

### Positive selection in full length *γ-c* shearwater OR genes

**Figure 4:**
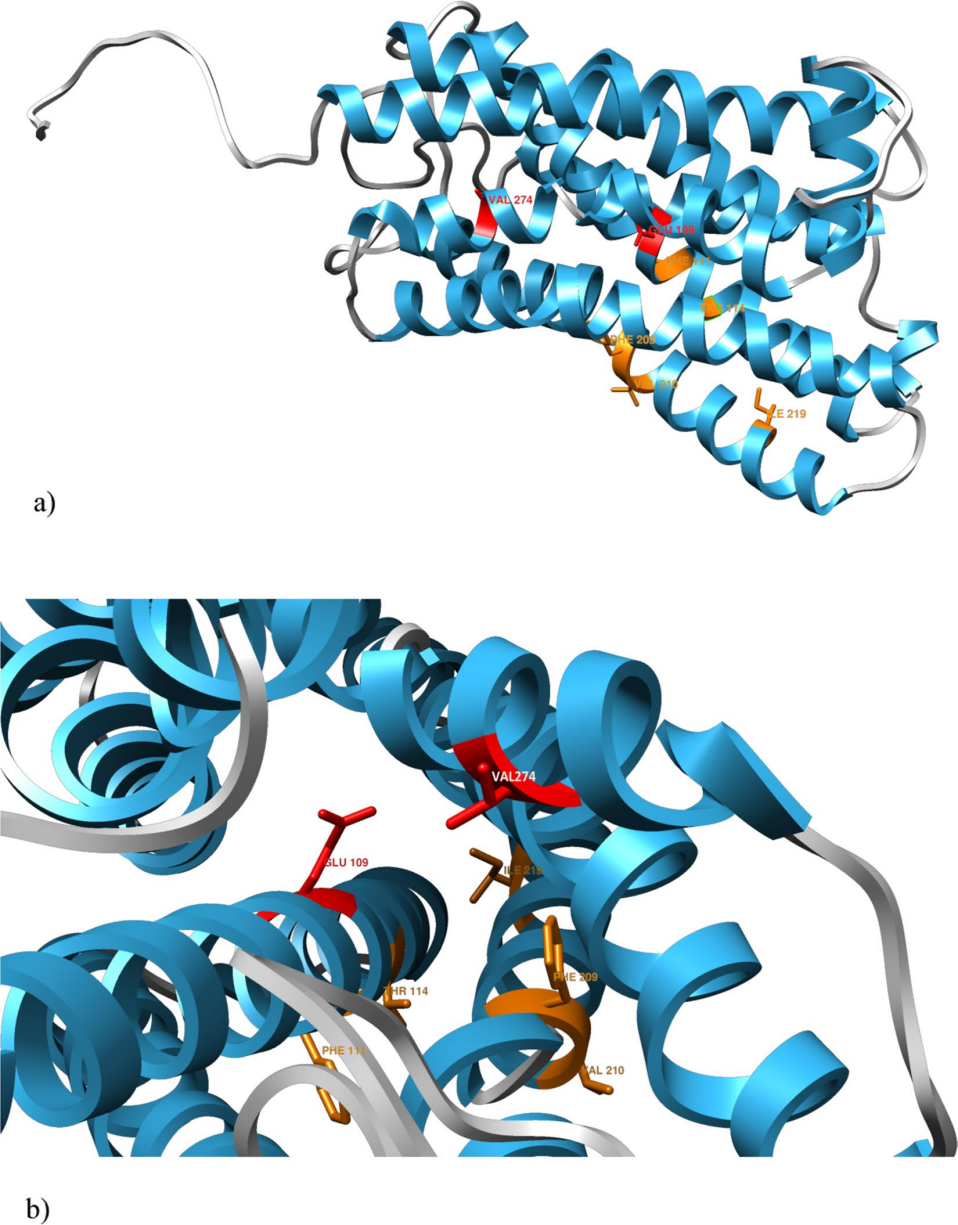
Secondary structure of a full-length *γ-c* OR gene from Cory’s shearwater. (**a**) Structure showing the α-helices of the trans-membrane domains in blue. The amino-acid sites estimated to be evolving under positive selection are plotted, showing two predicted by all methods to be under positive selection (in red) as well as those predicted by at least two methods to be under positive selection (in orange). (**b**). Detail of the secondary structure of the same OR receptor, showing the side chain of the two amino-acids with the strongest signal of positive selection, aa109 (TM3 domain) and aa274 (TM7 domain) (in red), orienting towards the center of the receptor, suggesting they might form part of the ligand binding pocket.

To determine the selection regimen governing the evolution of the intact *γ*-c shearwater genes, we considered two data partitions as GARD found evidence of one breakpoint at alignment position 246, which falls in the putative TM6 domain (Fig. 2). Based on the two-partition dataset of all full-length OR genes, the different methods found that the overall *d*_N_/*d*_S_ was never significantly different from 1, varying between *ω* = 0.552 (SLAC) and *ω* = 1.044 (REL). However, positive selection was identified on individual site codons of the full-length shearwater *γ-c* genes, consistent with a key role of selection in the functional diversification of these genes in the shearwater (Fig. 4a, Table 2). The number of sites with evidence of positive selection varied with the method used, with amino-acid positions 109 and 274 (respectively in TM3 and TM7 domains) identified by all individual methods as well as by the integrative analysis (Table 2). In addition, positive selection was identified in amino-acid positions 111, 114, 209, 210 and 219 by at least two methods. These five sites are also located in TM domains (Fig. 4a). Critically, amino acids 109 and 274 have side chains oriented towards the center of the receptor (Fig. 4b), suggesting that, in this species, they may be part of the odorant binding pocket.

**Table 2:** Positive selected sites detected by different likelihood approaches, as implemented in HyPhy.

# of gene sequences	*SLAC*^*†*^	*FEL*^*†*^	*REL*^*Ψ*^	*MEME*^*†*^	Integrative analysis
18	**109**, **274**	6, **109**, 111*, 114, 209*, 219*, **274**	107, **109**, 111*, 130, 157, 207, 209*, 210, 219*, 258, **274**	99, 103, **109**, 111*, 114, 154, 163, 209*, 210, 219*, 232, 259, 260, 261, 273, **274**, 311, 314	6, 103, 107, **109**, 111*, 114*, 130, 157, 207, 209*, 210*, 219*, 258 **274**, 314

Sites detected by all methods are underlined and shown in bold, those detected by two or more methods are shown by an asterisk. All methods were used on two alignment partitions due to breakpoint at codon site 264, as estimated by *GARD*.

^†^Significance level considered at *P* < 0.1 (Pond & Frost 2005b);

^Ψ^Bayes factor >50.

## Discussion

This work provides one of the first detailed molecular insights into the evolution of chemoreception in an avian group for which olfaction has been shown to play a vital role, by specifically studying sequence diversity and evolution of olfactory receptors in the Cory’s shearwater genome.

Several studies suggest that mammalian and avian OR repertoires are influenced by life histories^6,10,12,18–20^. Procellariiform seabirds have anatomical characteristics that suggest a strong reliance on a functional olfactory system, such as one of the largest olfactory bulb size to brain size (OB) ratio among extant avian species and specialized tube-like nostrils^42^. It has been suggested that there is a positive correlation between OB ratio, OR gene repertoire and olfactory function^10,15,21,25,28,29^. In this context, one might expect shearwaters to have more OR genes than species with smaller OB ratio and less obvious reliance on olfaction for survival, such as the chicken and zebra finch, in which 674 and 688 isolated OR genes have been identified, respectively^9^. However, the procellariiform Snow petrel was found to have among the lowest estimated number of OR genes (n = 212) among nine surveyed avian species, despite its much larger OB ratio^25^. Likewise, the Northern fulmar was found to only have an average number of OR genes in a genomic survey that included 48 phylogenetic diverse avian lineages^6^. The evidence from Cory’s shearwater is inconclusive in this regard. While we identified with certainty 220 OR genes (similar to the numbers found in other petrels but about a third of the total number estimated in the chicken), our study was limited by the maximum number of cosmid clones considered for sequencing, and possibly by the specificity of the probes used to screen the library, which were restricted to known OR motifs. Furthermore, putative OR sequences mapping to the edge of assembly contigs and for which the translated sequence was shorter than 75 amino acids were discarded. Finally, the 220 shearwater OR genes were identified in only a very small (albeit biased toward OR-containing) fraction of the genome (7.4 Mb) compared with the other species for which the whole genome was analyzed. On the other hand, we found 17 intact *γ-c* genes in the shearwater, a larger number than the 2 previously found in the fulmar, despite possibly having missed characterization of the full repertoire. This raises the possibility that a smaller olfactory receptor repertoire is compensated by a larger number of intact genes, or possibly a broader receptor binding pocket specificity through the use of other sensory receptors ^27,43^. This issue should be revisited once a high quality assembled Cory’s shearwater genome becomes available.

A remarkable finding of this study is that the vast majority (85%) of the OR genes identified in the shearwater belong to the *γ-c* clade. If the proportion of *γ-c* genes among all identified shearwater OR genes is representative of their real frequency in the genome, then the olfactory receptor family in this species is mostly composed of young paralogous loci, reflecting a relatively recent expansion of highly similar genes putatively linked to the recognition of volatile odorants. Although the *γ-c* clade seems to be as abundant in the shearwater as in the chicken (63%) and the zebra finch (80%), it was found to be not as abundant in other avian species, including the Northern fulmar^6^, although low genome assembly quality may have compromised the ability to characterize large expansions of recently duplicated genes in some species^44^. In two falcon species the *γ-c* clade was found missing, possibly reflecting reliance on other sensory cues^26^. Nevertheless, the presence of this striking *γ-c* diversity in the shearwater genome, and its higher proportion of intact genes relative to what has been found in most species surveyed so far^16^, is an example of how ecological adaptation is likely contributing to an enhanced olfactory function in Procellariiformes.

Lineage-specific duplications and losses of olfactory receptor genes is characteristic of the evolution of the OR gene family in vertebrates and have resulted in a large range of OR gene repertoire size across lineages^5,10–12,20^. The co-occurrence of *γ-c* intact, partial and pseudogenes in the shearwater OR repertoire, sometimes in the same contig, agrees with the extreme dynamic nature of the evolutionary models proposed to govern the genes encoding OR and other vertebrate multi-gene families^14,44,45^. In the context of the olfactory receptor evolution, the presence of many paralogs facilitates the evolution of novel gene functions that allow the recognition of new odors, under a scenario of neo-functionalization, but may also result in large number of pseudogenes due to functional redundancy and consequent relaxation of selective constraints^45–47^.

A hallmark of the olfactory receptor structure is a highly variable pattern of amino acid sequence conservation determining the identity and specificity of odorants that are recognized by each receptor. Although it is possible that some ligand interactions will be mediated by conserved amino acids^48,49^, peaks of polymorphism have been consistently detected on the same transmembrane domains in different species, namely in the TM3, TM5, TM6 and TM7^16^, similar with what was found in this study. If these domains do indeed form part of the odorant binding pocket in seabirds, as it has been shown in other vertebrate lineages^22^, it is not surprising to find evidence of diversifying selection at these sites, since allelic diversity would confer a selective advantage by allowing individuals to recognize a wider spectrum of odors.

Lineage-specific patterns of OR gene diversity have been found to be tightly linked to the habitat and foraging ecology of the different groups^6,18,21^. Procellariiform seabirds are pelagic species that forage over vast areas of a seemingly featureless sea expanse. However, these species rely on extremely fine tuned sensory cues to recognize heterogeneity in the marine environment, ensuring foraging efficiency over areas of patchy productivity. Exactly what odors are used to build an odoriferous seascape remains to be studied, but the large expansion of the *γ-c* clade, with its high levels of polymorphism and underlying patterns of adaptive evolution may contribute to the necessary discriminatory power to be used in a genetic mechanism linking the olfactory system with navigation, foraging and homing and perhaps even with kin recognition and other critical social interactions in this and other avian systems.

Much remains to be learnt about the structure and functioning of sensory receptors in birds^6,11,12,16^, and the isolation and characterization of olfactory receptor loci in the Cory’s shearwater is a significant step towards understanding the role of this fascinating multi-gene family in birds and, more generally, in the genomic underpinnings of ecological adaptation.

## Materials and Methods

### Sampling and OR gene isolation

A blood sample from a female Cory’s shearwater was collected in Selvagem Grande Island, and kept in 95% ethanol until laboratory analyses. Genomic DNA (~1500 μg) was extracted with a phenol-chloroform standard protocol and used to build a cosmid library (built with SuperCos1 Cosmid Vector Kit, Stratagene, La Jolla, CA, USA, outsourced to America Pharma Source LLC). The library had inserts that varied in size between 30–42 Kb and a titer equivalent to 4X of estimated genome size. Since there is no genome size estimates for Procellariiformes, we assumed 1,36 Gb based on the average of all avian genome sizes available^50^, a value similar to the genome assembly size for Northern fulmar of 1,135Gb^40^. Cosmid clones were screened using Southern Blotting with three probes, two targeting primarily sequences from the *γ-c* OR clade and one sequences from the non *γ-c* OR clade^25^. However, it is noteworthy that all probes targeted highly similar, evolutionarily conserved coding motifs that are present across all OR gene families, as implied by the cross-hybridization results below. Probes were generated as described elsewhere^25^. A ~ 500 bp fragment was amplified in the shearwater, which was cloned and sequenced to confirm olfactory receptor identity (Supplementary Info). We picked 96 positive clones, approximately a third were positive for all three probes, and the remaining for either one or two probes.

### Sequencing, assembly and annotation of OR gene-positive cosmids

The sequencing strategy aimed at generating long assemblies while minimizing costs and avoiding chimeras. Sanger sequencing generated ~ 900 bp long reads from cosmid ends which anchored each cosmid sequence. Depth of coverage was obtained from an equi-molar pool of non-tagged cosmids, using Illumina HiSeq 2000. 34 million Illumina 100 bp-long reads were used to build multiple assemblies at different depth of coverage using *Velvet*^51^. The best Illumina data assembly, with an estimated 139X coverage, consisted of 5,180 contigs (125 bp to 38 Kb in length), with a total cumulative length of 3.02 Mb (Supplementary Info). Guided by the Illumina data, the 96 cosmid clones were pooled into ten subsets that minimized within pool clone similarity. For each pool, DNA was sheared to 3Kb and a MID 454 GS FLX Titanium Paired End library was built and sequenced. A total of 541,133 reads were obtained for the 10 pools, with an average read length of 311 bp, resulting in an additional 20X coverage of each of the 96 cosmids. Hybrid assemblies were built with the *Celera Assembler* v.7^52^ using all 454 data and varying numbers of Illumina reads. Further analyses proceeded on the most comprehensive hybrid assembly.

All scaffold and degenerate contigs (the latter contain repetitive sequences that could not be unambiguously assembled with the main scaffolds) were processed through the Institute for Genome Sciences’eukaryotic structural annotation pipeline, which was configured to use a combination of tools, including *ab initio* gene finders, protein aligners and model combiners. Gene finders including *GeneMark*-*ES* (*GM*-*ES*)^53^, *Augustus* v2.7^54^ and *Maker*^55^ were run using chicken evidence data, and *GeneID* v1.4^56^ and *GlimmerHMM* v3.0.1^57^ used human and zebra finch informant data, respectively. *Exonerate*’s *protein2genome* option^58^ was run with a score cutoff of 500 and a cutoff of 75% to identify homologs to known chicken (n = 37,258 protein models) and zebra finch (n = 17,773) proteins. Finally, *Evidence Modeler* (*EVm*) r2012-06-25^59^ was used to combine evidence from spliced protein alignments and *ab initio* gene predictions into weighed consensus gene models.

Proteins were searched with Hidden Markov Models (HMMs) generated with *HMMbuild* using default settings and calibrated with *HMMcalibrate*. One HMM was built from full length OR genes (mostly from *G. gallus* and *T. guttata*), and the second was based on an alignment of partial OR gene sequences from eight different avian species^25^. The HMMs were validated by searching UniProt (Universal Protein Resource) using *HMMsearch* (EMBL_EBI) with a cut off value of 1e^−10^. A set of open reading frames (ORFs) was generated with ENSEMBL’s *getorf* which was searched using the new HMMs, with default settings. ORFs and *EVm* models matching HMMs were compared and uniqued, and the assembly location of each of the resulting genes determined. Proteins generated by *EVm* were annotated with a custom functional annotation pipeline, implemented within a reusable Ergatis pipeline, where input proteins are searched using several tools in parallel including *HMMer3*^60^ *NCBI-BLASTP* (cutoff 1e-6), *SignalP*^61^, *ScanProsite*^62^ and *TMHMM*^63^. The HMM database used for *HMMer3* is custom-made with TIGRFAMs, PFAM, among other custom records. Proteins which match the HMMs above trusted cut off levels inherit these annotations, or else BLAST results were used.

### Phylogeny of shearwater OR genes

Phylogenetic trees were estimated for two different amino acid (AA) alignments, one including all OR shearwater sequences of length > 75AA (n = 220) (Supplementary Dataset 1) and the other only the full-length ORs (n = 20) (Supplementary Dataset 2). The alignments included complete OR sequences from chicken, zebra finch and green anole (*Anolis carolinensis*) from^9^ and all non-redundant Northern fulmar (*Fulmarus glacialis*) proteins from Protein database of GenBank. We use a non-OR rhodopsin-like family GPCR gene (Adenosine receptor A2b from the chicken, zebra finch and emperor penguin (*Aptenodytes forsteri*), respective GenBank accession numbers NP_990418, XP_002198489, XP_009276449) as outgroup^21^. Sequences were aligned with MAFFT 7.12^64^, using E-INS-i parameters, and the alignments were manually curated in Mesquite v.2.75^65^. Approximate maximum-likelihood trees were reconstructed with FastTree v2.1.10^66^ under the JTT evolutionary model^67^, implemented in the CIPRES Science Gateway^68^. Nodal support was evaluated with Shimodaira-Hasegawa (SH) tests with 1,000 bootstrap replicates^66^.

### Estimates of positive selection on clade *γ*-c

The ratio of non-synonymous to synonymous substitutions (*ω* = *d*_N_*/d*_S_) is used as an indicator of the strength of selection on protein-coding genes. The presence of recombination or gene conversion, frequent in multi-gene families, precludes the use of methods that estimate selection based on a single phylogenetic tree^69,70^. We estimated *d*_N_*/d*_S_ per codon with four codon-based maximum likelihood methods available in HYPHY^71^, as implemented in the *Datamonkey* webserver^72^. We accounted for the possibility of recombination using the GARD module (also implemented in *Datamonkey*^72,73^ to infer breakpoints in the alignment and generate tree topologies for each of the non-recombinant data partitions. We then used SLAC, FEL, REL^70^, MEME^74^ methods, which differ in assumptions for the distribution of rates across sites or lineages, and an integrative approach, which considers all codon sites detected by each method, to infer selection within each partition. A significance level of *P* < 0.1 was used for SLAC, FEL and MEME, and a Bayes factor >50 for REL^70^.

*WEBLOGO* (http://weblogo.berkeley.edu/logo.cgi, ^75^) was used to generate a sequence logo with the amino acid alignment of all *γ-c* complete shearwater olfactory receptor sequences, to facilitate visualization of sequence variation for the putative transmembrane (TM), intracellular (IC) and extracellular (EC) domains. The height of each letter corresponds to the relative frequency of each amino acid at a given position, reflecting sequence conservation.

*Chimera*^76^ (http://www.cgl.ucsf.edu/chimera/index.html) was used to display the location of the polymorphic sites on the secondary structure of a shearwater complete *γ-c* OR.

### Ethical statement

The volume of the blood sample collected from the female shearwater was 150 μl (approximately 0.2% vol/weight). Sampling guidelines were approved by the Instituto da Conservação da Natureza e da Biodiversidade (ICNB) and by the Parque Natural da Madeira (currently Instituto das Florestas e Conservação da Natureza), Portugal under permit: 2/2012S. Sampling further followed the requirements of the Directive 2010/63/EU of the European Parliament and of the council for the protection of animals used for scientific purposes.

## Supplementary information

Supplementary information description.

Supplementary information.

Dataset 1.

Dataset 2.

## Data Availability

Cory’s Shearwater cosmid library shotgun Illumina HiSeq and 454 data as well as the assembled scaffold and degenerate contigs and OR gene sequences will be deposited in NCBI under BioSample accession number SAMN13721836. For additional information contact corresponding author (MCS).
