# Supplementary material for: Signature of adaptive evolution in olfactory receptor genes in Cory’s Shearwater supports molecular basis for smell in procellariiform seabirds
Source: Sci Rep. 2020 Jan 17;10:543. doi: 10.1038/s41598-019-56950-6 (PMC6969042; doi:10.1038/s41598-019-56950-6)
Supplement: Supplementary file 1 — Supplementary information description. [file 41598_2019_56950_MOESM1_ESM.pdf]

Supplementary information

Supplementary Figure 1. Contig size distribution (in bp) of only the Illumina reads (a) and scaffold size from the best final hybrid assembly of the 96 cosmids (b), including main scaffolds (with a cumulative length of 2.6Mb), and 22k degenerate scaffolds (with a cumulative length of 4.8Mb).

Supplementary Info S1. Probes and primer sequences used to survey the library for OR gene containing cosmid clones. The first two probes target the γ-*c* clade, the third probe target genes from the γ clade[25].

Supplementary Dataset S1. Amino acid sequence alignment of OR sequences from shearwater (n=220), fulmar, chicken, zebra finch, lizard and outgroups used to build the phylogenetic tree shown in Figure 3a.

Supplementary Dataset S2. Amino acid sequence alignment of OR sequences from shearwater (n=20), fulmar, chicken, zebra finch, lizard and outgroups used to build the phylogenetic tree shown in Figure 3b.
