# Supplementary material for: Signature of adaptive evolution in olfactory receptor genes in Cory’s Shearwater supports molecular basis for smell in procellariiform seabirds
Source: Sci Rep. 2020 Jan 17;10:543. doi: 10.1038/s41598-019-56950-6 (PMC6969042; doi:10.1038/s41598-019-56950-6)
Supplement: Supplementary file 2 — Supplementary information. [file 41598_2019_56950_MOESM2_ESM.pdf]

Supplementary information

Signature of adaptive evolution in olfactory receptor genes in Cory's Shearwater supports molecular basis for smell in procellariiform seabirds

Mónica C. Silva[1, *], Marcus Chibucos[2], James B. Munro[2], Sean Daugherty[2], M. Manuela Coelho[1], Joana C. Silva[2,3]

[1]Centre for Ecology, Evolution and Environmental Changes, Faculdade de Ciências, Universidade de Lisboa, 1749-016 Lisboa, Portugal

[2]Institute for Genome Sciences, University of Maryland School of Medicine, Baltimore, USA

[3]Department of Microbiology and Immunology, University of Maryland School of Medicine, Baltimore, USA

*corresponding author: mssilva@fc.ul.pt

a)

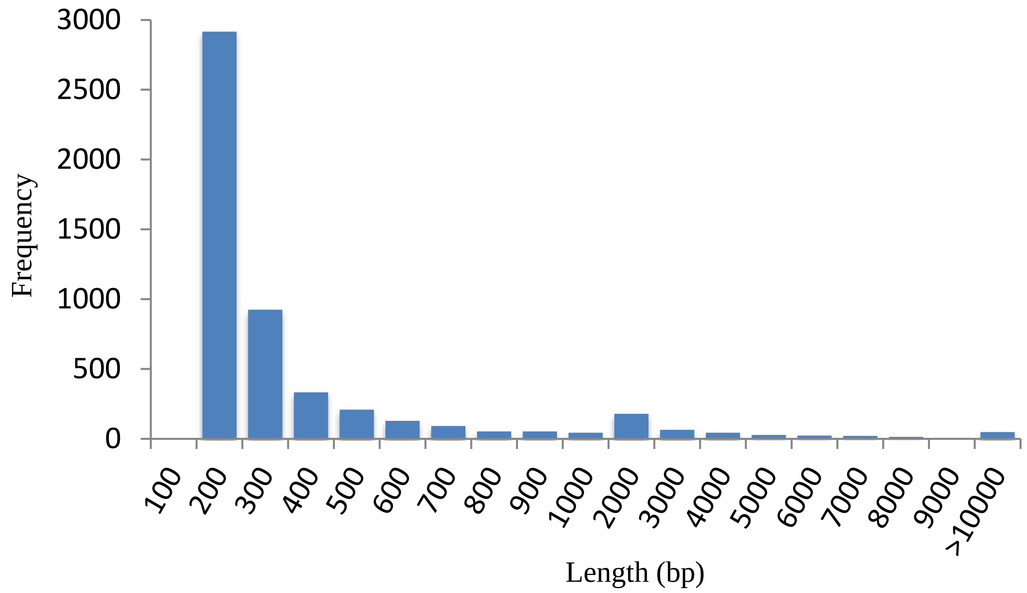

b)

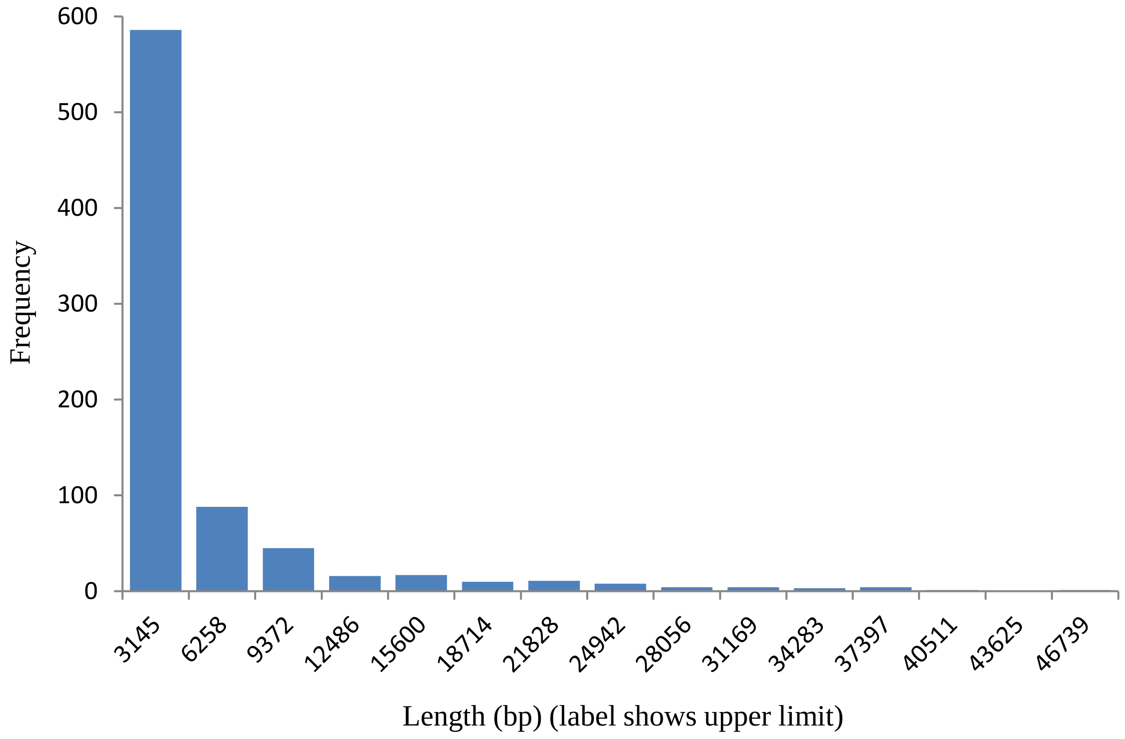

Supplementary Figure 1. Contig size distribution (in base pairs) of only the Illumina reads (a) and scaffold size from the best hybrid assembly of the 96 cosmids (b).

>Probe1
TGTTGCGATTTGTAAGCCCCTACACTACGGGACCCTCCTGGGCAGCAGAGCTTGTGTCCACATGGCAGC
AGCTGCCTGCGGCAGTGGGTTTATCATTGCTCTGCTGCACACTGCCAATACATTTTCATTGCCCCTCTGC
AAGGGCAATGCTGTGGACCAATTCTTCTGTGAAATTCCCCCACTCCTCAAGCTCTCCTGCT
CACACGCCTACCTCAGCGAAATTGGGCTTCTAGTGGTTACTTTTTCTTTAGGATTTGGGTGTTTTGTGTT
CATTGTGGTGTCCTATGTGGAGATCTTCAGGGCTGTGCTGAGGATCCCCTCTGAGCAGGGGTGGCACA
AAGCCTTTTCCACGTGCCTCCCTCACCTGGCCGTGGTCTCCCTGTTTGCCAGCACTGTCGTG
TTTGCTAACCTGAAGCCCCCCTCCATCTCCACCATATCCCTGGATCTGGTGGTGAGTGTTCTGTACTCAG
TGGTACCTCCAGCAGTCAACCCCTTCATATACAGCCTA

>Probe2
TGCGATTTGCAAGCCTCTACACTATGGGACCCTCCTGGGCAGCAGAGCGTGTGTCAGCATGGCAGCAG
CTGCCTGGGGCAGTGGGTTTCTCAATGCTGTGCTGCACACTGCCAATACATTTTCACTGCCCCTCTGCAA
GGGCAATGCTGTGGACCAGTTCTTCTGTGAAATCCCACAGATCCTCAAGCTCTCCTGCTCACACACCTAC
CTCAGGGAAGTTGGGCTTATAGAGTTTAGCCTTTCAGTAGCTTTTGGGTGTTTTATTTTCATCGTGCTGT
CCTATGTGCATATCTTCAGGGCTGTGTTGAGGATCCCCTCTGAGCAGGGGTGGCACAAAACCTTCTCCA
CGTGCCTCCCTCACCTGGCCGTGGTCTCCCTGTTTGTCAGCACTGCCATGTTTGCCTACCTGAAGCCCCC
CTCCATCTCCTCCTCATCCTTGGATCTGGTCGTGACAGTTCTTTACTCAGTGGTACCTCCAGCGGTGAAC
CCCTTCATCTACTCCCTA

>Probe3
TGCGATGGCGTACGACCGGTACATTGCGATATGCAAACCATTGCATTACACAACCATAATGAACGGCA
GGTGCTGCCTCCAGTTAGCAGGTGGATCCTGGATAAATGGGTTGTTGGCTAGTGCCATAGTCACCTCTC
TGATGCCACAGCTAACTTTCTGTGGCCCAAATGAAATTGATCATTTCTTCTGTGACTGCACACCGGTGGT
AAAGCTCTCCTGCAGTGATACCCACTGGATTGAGCTTGTAACATTCATCTTGGCCTCCCTTTTCACACTAC
CCCCATTTCTCTTGACGCTCATATCTTACGTGTACATCAGCAACACTGTCCTCAGGATCCCATCCTCCACA
GGAAGGCATAAAGCTTTTTCTACCTGTTCTTCCCACCTCACTGTGGTGACACTTTTCTACGGGACCCTGA
TTGTTGTGTACTTGGTACCAAAACCACACACGCTGGGAGACCTCAACAAAGTATTCTCTGTCTGCTACAC
AGTTCTCACTCCCATGCTCAACCCCCTCATCTA

PROBE 1
Primer forward:
ICKPLHY - 5'-ATCTGYAARCCIYTICAYTA
Primer reverse:
NPFIYS(F/L) - 5'-ARISWRTADATRAAIGGRTT

PROBE 2
Primer forward:
VAICKPLHY - 5'-RTTGCIATYTGYAARCCYCTRCACTA
Primer reverse:
NPFIYS(F/L) - 5'-ARISWRTADATRAAIGGRTT

PROBE 3
Primer forward:
AMAYDRY - 5'-GCIATGGCITAYGAYMGITA

Primer reverse:
PMLNPLIY- 5'-TADATIAGIGGRTTIAGCATIGG

Supplementary Info S1. Probes and primer sequences used to survey the library for OR gene containing cosmid clones. The first two probes target the γ-c clade, the third probe target genes from the γ clade. Primers from Steiger S.S., Fidler A.E., Valcu M., Kempenaers B. Avian olfactory receptor gene repertoires: evidence for a well-developed sense of smell in birds? Proc Biol Sci 275, 2309-2317 (2008).
